# Xuan Bai Cheng Qi formula as an adjuvant treatment of acute exacerbation of chronic obstructive pulmonary disease of the syndrome type phlegm-heat obstructing the lungs: a multicenter, randomized, double-blind, placebo-controlled clinical trial

**DOI:** 10.1186/1472-6882-14-239

**Published:** 2014-07-11

**Authors:** Miao Liu, Xianggen Zhong, Yuhang Li, Fengjie Zheng, Ruohan Wu, Yan Sun, Jinchao Zhang

**Affiliations:** 1 School of Preclinical Medicine, Beijing University of Chinese Medicine, 11 East Third Ring Road, Beijing 100029, China

**Keywords:** Acute exacerbation, Chronic obstructive pulmonary disease, Xuan Bai Cheng Qi, Phlegm-heat obstructing lung, Syndrome, Traditional Chinese medicine

## Abstract

**Background:**

Acute exacerbation of chronic obstructive pulmonary disease (AECOPD) is a common cause of morbidity and mortality. Traditional Chinese medicine (TCM) is used to treat AECOPD as adjunctive therapy. This study aimed to evaluate the efficacy and safety of the TCM formula Xuan Bai Cheng Qi as an adjuvant therapy for AECOPD patients with the syndrome type of phlegm-heat obstructing the lungs.

**Methods:**

A multicenter, randomized, double-blind, placebo-controlled clinical trial was conducted. A total of 244 patients were divided into the intervention group (n = 122, treated with conventional medicine and Xuan Bai Cheng Qi) and the control group (n = 122, treated with conventional medicine and placebo). Total symptom scores (cough, phlegm, wheezing, chest congestion) before treatment and at 3, 5, 7, 10 days post-treatment were recorded. Lung function, arterial blood gas, serum inflammatory cytokines, oxidation/anti-oxidation index were observed before treatment and at the end of the 10-day treatment.

**Results:**

A total of 242 patients completed the study. The full analysis set (FAS) population was 244 and the per-protocol analysis set (PPS) population was 229. After the 10-day treatment, symptom scores of the Xuan Bai Cheng Qi group were significantly lower over time compared with the control group (FAS: mean difference -1.84, 95% CI -2.66 to -1.03, *P* < .001; PPS: mean difference -1.87, 95% CI -2.71 to -1.03, *P* < .001). FEV_1_, FVC, and FEV_1_%pred were significantly higher over time in the Xuan Bai Cheng Qi group compared with those in the control group (day 10, FAS and PPS: *P* < .05). PaO_2_ and PaCO_2_ were significantly improved in the Xuan Bai Cheng Qi group (day 10, FAS and PPS: *P* < .05). Xuan Bai Cheng Qi was also found to ameliorate cytokine levels and oxidation/antioxidant index compared with placebo. There were no differences in safety variables and adverse events between the two groups.

**Conclusions:**

Xuan Bai Cheng Qi formula appears to be a safe and beneficial treatment for AECOPD of phlegm-heat obstructing the lungs syndrome type.

## Background

Chronic obstructive pulmonary disease (COPD) is defined by irreversible or partially reversible airway obstruction in persons with chronic bronchitis or emphysema. Experts predict that COPD will become the fourth leading cause of death worldwide by 2030 [1].

Hastening of normal reduction in lung function that occurs with age and repeated exacerbations are key features of COPD. Acute exacerbation of chronic obstructive pulmonary disease (AECOPD) is a common cause of morbidity and mortality in COPD patients [2]. These exacerbations are associated with worsening respiratory symptoms and lung function [3]. Median time post-exacerbation to recovery of peak flow is 6 days and time to recovery from symptoms is 7 days [4].

Many etiologic factors contribute to COPD exacerbations such as continued smoking, respiratory tract infections, and exposure to particles from air pollution and the work environment. Upper respiratory tract infections are the most common triggering factor of COPD exacerbations. Thus, the management of exacerbations creates an enormous burden on health care services [5].

Traditional Chinese medicine (TCM) is used extensively in the treatment of AECOPD in Asia, particularly in China. The effects of TCM, especially herbal medicines, in improving respiratory symptoms and decreasing the incidence of COPD exacerbations are well known. One such herbal medicine is Xuan Bai Cheng Qi formula. In Chinese medicine, the primary syndrome type of AECOPD is phlegm-heat obstructing the lungs [6]. The actions of Xuan Bai Cheng Qi formula are to clear lung heat and dissipate phlegm, so for this reason it is commonly prescribed for AECOPD [7]. The remedy consists of four Chinese herbs: *Gypsum fibrosum* (gypsum), *Rheum officinale* Baill (rhubarb root and rhizome), *Armeniacae amarum* (apricot seed or kernel), and *Trichosanthes kirilowii* (trichosanthes peel) (Table 1). Xuan Bai Cheng Qi is a classical prescription dating to the Qing Dynasty (late 1700s) text *Systematic Differentiation of Warm Pathogen Diseases* by Wu Jutong. Our previous studies found that Xuan Bai Cheng Qi improves oxidant/antioxidant imbalance, pulmonary inflammation in the rat model of COPD [8,9]. Clinical studies have postulated that Xuan Bai Cheng Qi is effective as a supplemental remedy for treating AECOPD or respiratory failure [10,11]. However, due to the limited sample sizes and methodological problems apparent in these studies the evidence for the effectiveness of Xuan Bai Cheng Qi was not robust. Therefore, we performed a multicenter, randomized, double-blind, placebo-controlled clinical trial to evaluate the efficacy and safety of Xuan Bai Cheng Qi in the treatment of AECOPD of the syndrome type phlegm-heat obstructing the lungs.

**Table 1:** Medicinals in Xuan Bai Cheng Qi formula

**Chinese name (pinyin)**	**Latin name**	**English common name**	**Daily decoction dose**^**a**^**(g)**	**Dose per 1,000 g granule**^**b**^**(g)**
Sheng Shi Gao	*Gypsum fibrosum*	Gypsum	30	435
Sheng Dahuang	*Rheum officinale* Baill	Unprocessed rhubarb root and rhizome	18	261
Ku Xing Ren	*Armeniacae amarum*	Bitter apricot seed	12	174
Gua Lou Pi	*Trichosanthes kirilowii*	Trichosanthes peel	9	130

^a^Amount of each medicinal if formula were prepared as a decoction.

^b^Amount of each medicinal used in preparing granule form of this formula.

## Methods

### Ethics and trial registration

This study was approved by the research ethics committees at all participating centers and on file at Dongzhimen Hospital affiliated to Beijing University of Chinese Medicine (DZMSP20090302). This trial was registered with the Chinese Clinical Trial Registry (ChiCTR-TRC-09000533).

### Patients

#### Diagnostic criteria

AECOPD diagnosis was based on criteria established by the Global Initiative for Chronic Obstructive Lung Disease (GOLD, 2007 version) [12], and by the Chinese Society of Respiratory Diseases (2007 version) [13].

Diagnostic criteria for TCM syndrome of phlegm-heat obstructing lung was based on State Administration of Traditional Chinese Medicine guidelines [14] and criteria outline by Wang and Lu [15]. Primary symptoms were: cough and/or gasping for breath; yellow and viscous sputum. Accompanying symptoms were: irritability and restlessness; abdominal distension; thirst with desire for cool drink; fever without chills; yellow urine; constipation with hard stool; red tongue with yellow or yellow greasy coating; slippery and rapid pulse. Phlegm-heat obstructing the lungs was diagnosed when any two primary symptoms along with any two accompanying symptoms were present.

#### Inclusion and exclusion criteria

Persons of either gender were included in the study if they: met the diagnostic criteria of AECOPD; met TCM diagnostic criteria for syndrome of phlegm-heat obstructing lung; had symptoms defined by GOLD as level 1 to 4; were aged between 18 to 85 years; were within 5 days post-AECOPD; had not participated in other interventional trials in the previous 1 month; and were willing to participate in the study voluntarily and signed an informed consent.

Persons were excluded from the study if they had any of the following: airflow limitation due to bronchiectasis, cystic fibrosis, lung cancer, or other respiratory disease; co-morbidities such as acute heart failure, acute cerebral hemorrhage, gastrointestinal bleeding, aplastic anemia, or other severe or life-threatening diseases; unstable hypertension, coronary heart disease, or diabetes; mental disease, severe neurologic deficits or other disorders that impaired understanding or cooperation with the investigation; serious liver, kidney, or hematopoietic disease; recent use of immunosuppressive agents; known allergy to herbs or drugs used in the trial. Women planning to become or were pregnant or breastfeeding were also excluded.

#### Participant enrollment

Participants in the trial were inpatients diagnosed with AECOPD of the syndrome phlegm-heat obstructing the lungs. They were recruited and enrolled on a rolling basis and observed from October 2009 through January 2013. Eight teaching hospitals in China were a part of the trial: Dongzhimen Hospital affiliated to Beijing University of Chinese Medicine (40 cases; 16.4%); Dongfang Hospital affiliated to Beijing University of Chinese Medicine (26 cases; 10.7%); Shanghai Shuguang Hospital affiliated to Shanghai University of Chinese Medicine (45 cases; 18.4%); University Hospital of Gansu Traditional Chinese Medicine (10 cases; 4.1%); First Affiliated Hospital of Anhui University of Chinese Medicine (23 cases; 9.4%); Hebei Provincial Hospital of Chinese Medicine (38 cases; 15.6%); Henan Province Kaifeng City Hospital of Traditional Chinese Medicine (18 cases; 7.4%); Affiliated Hospital of Liaoning University of Traditional Chinese Medicine (44 cases; 18.0%).

### Treatments

#### Conventional therapy

All participants in the trial and control groups were given conventional treatment based on GOLD [12] and the Chinese Society of Respiratory Diseases [13]. Conventional medicine regimen: oxygen therapy (1–3 L/min) by nasal cannula or Venturi mask; open-label cefotaxime 2 g daily (iv q 12 h) for 10 days or an alternate antibiotic if the patient was allergic to or intolerant of cefotaxime (Table 2); inhaled long-acting β-agonist (either salmeterol or formoterol) and an inhaled long-acting anticholinergic bronchodilator (tiotropium) after randomization; if the patient was using an inhaled steroid or inhaled steroid/long-acting β-agonist combination product at the time of randomization, this medication was continued for patients in both groups; if the patient’s baseline FEV_1_ was <50% of predicted, 30–40 mg prednisolone per day for 7–10 days was administered. Supplemental salbutamol, ambroxol, and aminophylline were given as needed throughout the trial period.

**Table 2:** Application of antibiotics at each stage of AECOPD

**COPD stage of acute exacerbation**	**Microorganisms**	**Antibiotics**
GOLD stages I and II	*Haemophilus influenzae*, *Streptococcus pneumoniae*, *Moraxella catarrhalis*	Penicillin, beta lactamase/enzyme inhibitor (amoxicillin/clavulanic acid), macrolides (clarithromycin, roxithromycin, azithromycin), first generation (cefazolin, cefradine) or second generation cephalosporins (cefuroxime, cefaclor), doxycycline, levofloxacin.
GOLD stages III and IV; no risk factors for infection with *Pseudomonas aeruginosa*	*Haemophilus influenzae*, *Streptococcus pneumoniae*, *Moraxella catarrhalis*, *Klebsiella pneumoniae*, *Escherichia coli*, Enterobacteriacea	Beta-lactam/enzyme inhibitor (cefuroxime), second generation cephalosporins, fluoroquinolones (levofloxacin, moxifloxacin, gatifloxacin), third generation cephalosporins (ceftriaxone, cefotaxime).
GOLD stages III and IV; risk factors for infection with *Pseudomonas aeruginosa*	All above microorganisms plus *Pseudomonas aeruginosa*	Third generation cephalosporins (ceftazidime), cefoperazone/sulbactam, piperacillin/tazobactam, imipenem, meropenem, can also be combined with aminoglycosides, fluoroquinolones (ciprofloxacin).

*Abbreviations*: *AECOPD* acute exacerbation of chronic obstructive pulmonary disease, *COPD* chronic obstructive pulmonary disease, *GOLD* Global Initiative for Chronic Obstructive Lung Disease.

#### Experimental and control treatments

Participants in the intervention group received Xuan Bai Cheng Qi formula in granule form as an adjunct to conventional therapy (Table 1). Participants in the control group received conventional therapy plus placebo granules.

#### Manufacturing and administration of Xuan Bai Cheng Qi and placebo granules

Both Xuan Bai Cheng Qi granules and the placebo granules were produced and packaged by Jiang Yin Tian Jiang Pharmaceutical Co. Ltd. (Jiangsu, China) under good manufacturing practice regulations of China (Approval Number: 0905301–4).

Xuan Bai Cheng Qi granules were prepared by decocting the four herbs together, resulting in a concentrated liquid and extraction and capture of the volatile oils. The concentrated liquid was spray-dried on a starch base powder to obtain the final product. Each package of granules was 4.5 g. Quality of the granules was tested for consistency with industry standards.

The placebo granules were designed to taste, smell, and resemble the Xuan Bai Cheng Qi granules.

Participants were given packages of Xuan Bai Cheng Qi or placebo granules to prepare on their own over the 10-day trial period. Four packages were to be taken each day by dissolving the granules in 300 mL boiled water with 150 mL taken twice daily. To assess adherence, at the completion of the trial on day 10, participants turned in used and unused packages of granules to research team nurses.

At the start of the trial, participants were instructed not to receive other TCM therapy associated with the treatment of AECOPD.

### Outcome measures

A case report form (CRF) was designed by the research steering committee. Spot checks were conducted throughout the trial by seven independent reviewers from within the steering committee to check completion of the CRF and conformance to the protocol. Randomly selected CRFs were reviewed during the trial to ensure quality.

Primary outcome measure was symptom scores, which were assessed and recorded on day 1, 3, 5, 7, 10. Secondary outcome measures were lung function, arterial blood gas analysis, pro-inflammatory biomarkers (cytokines) and oxidation/antioxidant index, which were measured and recorded on days 1 and 10.

#### Symptom scores

A symptom score sheet was designed based on the typical format of a 4-point Likert scale [16] with questions pertaining to four AECOPD symptoms in terms of TCM syndrome of phlegm-heat obstructing the lung (cough, phlegm, wheezing, chest congestion) [17]. Members of the research team administered the questionnaire to participants. Severity of symptoms was assigned the following points: 0 (none), 2 (mild), 4 (moderate), and 6 (severe). Total score range was from 0 (asymptomatic) to 30 (severe).

#### Lung function

Lung function was evaluated in participants using spirometry. Forced expiratory volume in one second (FEV_1_), forced vital capacity (FVC) and FEV_1_ percentage of the predicted value (FEV_1_% pred) were tested.

#### Arterial blood gas analysis

Arterial blood gas parameters that were measured were pH, arterial partial pressure of oxygen (PaO_2_), arterial partial carbon dioxide pressure (PaCO_2_).

#### Pro-inflammatory biomarkers (cytokines)

Levels of tumor necrosis factor alpha (TNF-α), interleukin-4 (IL-4), interleukin-8 (IL-8), interleukin-1 beta (IL-1β), interleukin-6 (IL-6), interleukin-2 (IL-2) in serum were quantified using an enzyme-linked immunosorbent assay (ELISA) kit (BD Biosciences, San Diego, CA).

#### Oxidant/antioxidant balance

Levels of superoxide dismutase (SOD) and malondialdehyde (MDA) in serum were quantified using commercially available kits (Nanjing Jiancheng BioEngineering Institute, Nanjing, China).

#### Safety variables

Complete blood count, urine, liver and kidney function tests, and electrocardiography were performed pre- and post-treatment (days 1 and 10). Any adverse events that occurred during the treatment period were recorded.

#### Adherence

Treatment adherence was assessed by investigators at each center counting used and unused granule packages.

### Study design

This was a multicenter, randomized, double-blind, placebo-controlled, parallel group clinical trial.

#### Sample size

As an exploratory study, reference power calculations were lacking as there have been only a few small-size trials in China of Xuan Bai Cheng Qi formula for treatment of AECOPD. Therefore, we inferred data from literature on TCM therapies with similar efficacy as Xuan Bai Cheng Qi that treated the same syndrome of phlegm-heat obstructing the lungs.

The approximate calculation of sample size was determined based on the effective rate of symptom scores (= (total scores of baseline - total scores of end)/total scores of baseline × 100%) [18]. The effective rate of conventional medicine plus placebo (75%) and the effective rate of conventional medicine plus Xuan Bai Cheng Qi (90%) were based on comparable studies [19,20]. The control group and trial group adopted 1:1 parallel design. With 5% one-sided significance level and 90% statistical power, it was determined that 122 participants should be randomly assigned to each group, considering an approximate 10% drop-out rate.

#### Randomization and blinding

Participants were randomly allocated to receive Xuan Bai Cheng Qi formula or placebo. Randomization was achieved using SAS 8.0 software to generate random numbers. Simple randomization was used, with each block comprised of eight participants. Randomization numbers were assigned to participants in chronological order after their screening assessments. Participants in the active arm received Xuan Bai Cheng Qi formula (granules) plus conventional therapy. Participants in the inactive arm received conventional therapy plus matching placebo.

To ensure allocation concealment, opaque and sealed emergency letters were printed according to randomized list. In the event of an emergency, the participant’s randomization code and group allocation could be identified by the emergency envelope, which were on file at all centers. Third-party investigators were assigned in each research center as the contact person who preserved and recorded the randomization information. They did not have contact with other trial investigators, therefore, they did not affect enrollment or randomization.

Xuan Bai Cheng Qi and placebo granules were re-packaged in identical wrappers at each center before distribution to participants, and numbered sequentially according to randomization schedule. Outcome assessments were made by an independent statistician who was blinded to group allocation, intervention, and trial management and did not take part in any other part of the trial. Both participants and investigators were blinded until the end of study.

### Statistical analysis

All data were entered with Epidata 3.0 software (The EpiData Association, Odense, Denmark) by two independent data recorders. All randomized patients were included in data analysis. Full analysis set (FAS) was used to analyze the baseline data and outcome data of the cases that went through randomization and received treatment. Each participant underwent at least one time-point observation during the trial. Partially missing data were imputed with the use of the last-observation-carried-forward (LOCF) method, whereby missing values were replaced by the last non-missing value. Per-protocol analysis set (PPS) was used to analyze the evaluation data of cases that completed the trial without major protocol deviations.

Continuous variables were analyzed using repeated-measures analysis of variance (ANOVA) and/or analysis of covariance. Mauchly’s test was used to assess sphericity in the repeated measures ANOVAs, and the Greenhouse Geisser correction was applied to the degrees of freedom (DFs) if necessary. Main effects for time, for the interaction between time and the intervention group, and for the overall difference between groups (Xuan Bai Cheng Qi versus placebo) were assessed. Categorical variables were analyzed using chi-square test, Fisher’s exact test, or Cochran-Mantel-Haenszel test. The effect size estimate and 95% confidence interval (CI) were applied for comparing the treatments. All statistical procedures were performed with SPSS 17.0 software (SPSS Inc, Chicago, IL).

### Data monitoring committee

A data monitoring committee at Dongzhimen Hospital comprised of clinicians, statisticians, epidemiologists oversaw data analyses and ensured protocol adherence and safety of the trial. Data were reviewed and monitored every 6 months.

## Results

### Study population

**Figure 1:**
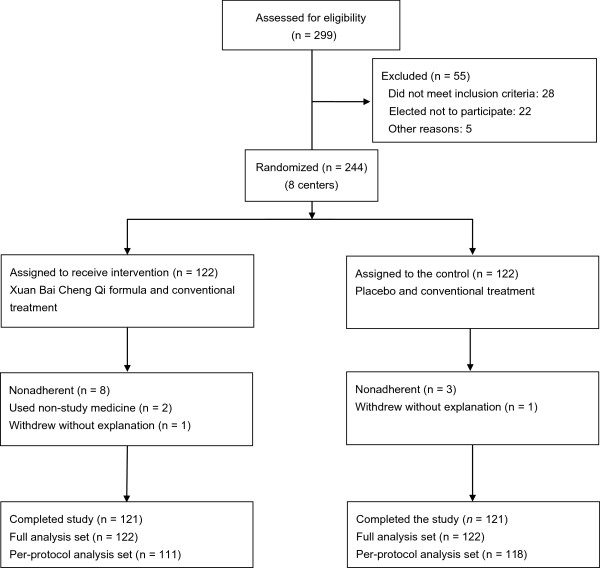
Trial profile.

A total of 299 COPD patients were assessed for eligibility in the trial. After 55 patients were excluded due to not meeting eligibility criteria, 244 patients were enrolled and underwent randomization (Figure 1). Two participants dropped out on day 7 without explanation, but they received treatment and also completed one time point observation. Thus, a total of 242 evaluable participants were in the study, with 121 in the Xuan Bai Cheng Qi group and 121 in the control group. The FAS population was 244, with 122 in the trial group and 122 in the control group. Eleven participants were protocol noncompliant, and two participants used non-study medications, which affected outcome calculation. Ultimately, PPS population was 229, with 111 in the trial group and 118 in the control group.

In terms of demographic and baseline characteristics of participants (Table 3), there were no significant differences between the Xuan Bai Cheng Qi and control groups in gender, age, course of disease, body mass index (BMI), exacerbations, FEV_1_/FVC, and GOLD classification of lung function.

**Table 3:** Baseline characteristics of participants

	**XBCQ (n = 122)**	**Control (n = 122)**
Age (SD), y	70.1 ± 9.8	70.7 ± 9.8
Body mass index (SD), kg/m^2^	23.36 ± 3.44	22.76 ± 3.11
Gender (male/female)		
Male (n) %	88 (72.1%)	82 (67.2%)
Female (n) %	34 (27.9%)	40 (32.8%)
Body temperature (°C)	36.68 ± 0.56	36.66 ± 0.58
Exacerbation		
Frequency (times)	2.8 ± 1.28	2.6 ± 1.24
Currently exacerbation	3.5 ± 1.36	3.5 ± 1.29
Smoking status		
Currently smoking, n (%)	22 (18.03%)	16 (13.11%)
Never smoked, n (%)	54 (44.26%)	52 (42.62%)
Ever smoked, n (%)	46 (37.70%)	54 (44.26%)
No. of packs/year	299.8 ± 170.0	337.9 ± 192.6
FEV_1_/FVC	55.02 ± 10.477	56.91 ± 9.592
GOLD classification		
GOLD stage I, n (%)	10 (8.20%)	9 (7.38%)
GOLD stage II, n (%)	56 (45.90%)	58 (47.54%)
GOLD stage III, n (%)	47 (38.52%)	45 (36.89%)
GOLD stage IV, n (%)	9 (7.38%)	10 (8.20%)

*Abbreviations*: *COPD* chronic obstructive pulmonary disease, *FEV*_1_ forced expiratory volume in one second, *FVC* forced vital capacity, *GOLD* Global Initiative for Chronic Obstructive Lung Disease, *SD* standard deviation, *XBCQ* Xuan Bai Cheng Qi.

### Symptom scores

ANOVA revealed a significant main effect of group (*P* = .001), a significant main effect of time (*P* < .001), as well as a significant group × time interaction (*P* < .001). Total symptom scores of the Xuan Bai Cheng Qi group were significantly lower over time compared with those of the control group (FAS: mean difference -1.84, 95% CI -2.66 to -1.03, *P* < .001; PPS: mean difference -1.87, 95% CI -2.71 to -1.03, *P* < .001). This difference was achieved within the first 3 days of treatment (FAS: mean difference -2.57, 95% CI -3.64 to -1.49, *P* < .001; PPS: mean difference -2.60, 95% CI -3.70 to -1.49, *P* < .001). At days 3, 5, 7, and 10, there were significant differences in total symptom scores of the Xuan Bai Cheng Qi group compared with those of the control group (Table 4).

**Table 4:** Total symptom scores in Xuan Bai Cheng Qi and control groups during 10 - day treatment

**Total symptom scores**	**Full analysis set**			**Per - protocol analysis set**		
	**XBCQ**^**a**^	**Control**^**a**^	**Treatment effect**^**b**^	**XBCQ**^**a**^	**Control**^**a**^	**Treatment effect**^**b**^
	**(n = 122)**	**(n = 122)**	**(95% CI;** ***P*-value)**	**(n = 122)**	**(n = 122)**	**(95% CI;** ***P-*** **value)**
1 d	19.70 (4.23)	20.10 (4.28)	-0.39 (-1.47, 0.68; .471)	19.64 (4.24)	20.09 (4.32)	-0.45 (-1.56, 0.67; .433)
3 d	13.92 (4,46)	16.48 (4.08)	-2.57 (-3.64, -1.49; < .001)	13.82 (4.50)	16.42 (3.95)	-2.60 (-3.70, -1.49; < .001)
5 d	10.64 (4.28)	13.20 (4.18)	-2.56 (-3.63, -1.49; < .001)	10.59 (4.24)	13.12 (4.10)	-2.52 (-3.61, -1.44; < .001)
7 d	7.72 (4.34)	9.64 (3.68)	-1.92 (-2.93, -0.90; < .001)	7.64 (4.38)	9.54 (3.65)	-1.90 (-2.95, -0.86; < .001)
10 d	5.21 (4.43)	7.00 (3.72)	-1.79 (-2.82, -0.76; 0.001)	5.03 (4.29)	6.92 (3.74)	-1.89 (-2.93, -0.84; < .001)
Mean^c^	11.44 (3.36)	13.28 (3.09)	-1.84 (-2.66, -1.03; < .001)	11.34 (3.40)	13.22 (3.05)	-1.87 (-2.71, -1.03; < .001)

*Abbreviations*: *CI* confidence interval, *XBCQ* Xuan Bai Cheng Qi.

Mauchly’s test of sphericity was applied: W = .506, *P* < .001.

^a^Data presented as mean (SD).

^b^Repeated-measures analysis of variance (ANOVA) for estimate with 95% confidence interval for difference.

^c^Mean = change from baseline.

### Lung function

FEV_1_, FVC, and FEV_1_ % pred were significantly higher over time in the Xuan Bai Cheng Qi group compared with the same parameters in the control group (FEV_1_, FAS: mean difference 0.14, 95% CI 0.06 to 0.22, *P* < .001, PPS: mean difference 0.13, 95% CI 0.06 to 0.21, *P* = .001; FVC, FAS: mean difference 0.17, 95% CI 0.06 to 0.28, *P* = .002, PPS: mean difference 0.16, 95% CI 0.05 to 0.27, *P* = .004; FEV_1_ % pred, FAS: mean difference 7.33, 95% CI 4.58 to 10.07, *P* < .001, PPS: mean difference 7.20, 95% CI 4.37 to 10.03, *P* < .001) (Table 5).

**Table 5:** Lung function measurements in Xuan Bai Cheng Qi and control groups before and after 10-day treatment

**Lung function**^**a**^	**XBCQ**^**b**^			**Control**^**b**^			**Treatment effect**^**d **^**(95% CI; *P *** **value)**
	**Day 1 (Baseline)**	**Day 10 (End)**	**Mean D**^**c**^	**Day 1 (Baseline)**	**Day 10 (End)**	**Mean D**^**c**^	
FEV_1_ (liters)							
FAS (122,122)	1.18 (0.41)	1.44 (0.49)	0.24 (0.29)	1.19 (0.40)	1.29 (0.47)	0.10 (0.31)	0.14 (0.06, 0.22; < .001)
PPS (111,118)	1.20 (0.42)	1.44 (0.49)	0.24 (0.29)	1.19 (0.39)	1.29 (0.47)	0.10 (0.31)	0.13 (0.06, 0.21; .001)
FVC (liters)							
FAS (122,122)	2.16 (0.62)	2.40 (0.67)	0.23 (0.38)	2.10 (0.58)	2.17 (0.62)	0.08 (0.48)	0.17 (0.06, 0.28; .002)
PPS (111,118)	2.17 (0.60)	2.40 (0.65)	0.23 (0.38)	2.11 (0.57)	2.19 (0.60)	0.08 (0.48)	0.16 (0.05, 0.27; .004)
FEV_1 _% pred							
FAS (122,122)	51.54 (19.49)	62.39 (19.57)	10.59 (11.76)	52.13 (19.17)	55.35 (20.59)	3.22 (10.53)	7.33 (4.58, 10.07; < .001)
PPS (111,118)	52.60 (19.74)	63.38 (19.36)	10.49 (11.84)	52.17 (19.27)	55.56 (22.68)	3.39 (10.50)	7.20 (4.37,10.03; < .001)

*Abbreviations*: *CI* confidence interval, *COPD* chronic obstructive pulmonary disease, *FEV*_1_ forced expiratory volume in one second, *FEV*_1_*% pred* FEV_1_ percentage of predicted value, *FVC* forced vital capacity, *GOLD* Global Initiative for Chronic Obstructive Lung Disease, *Mean D* mean difference, *XBCQ* Xuan Bai Cheng Qi.

^a^FAS population was 122 in the XBCQ group and 122 in the control group; PPS population was 111 in the XBCQ group and 118 in the control group.

^b^Data presented as mean (SD).

^c^*P* < .01: Change between pre-treatment and post-treatment (within group difference by paired t-test).

^d^Covariance analysis estimate with 95% confidence interval for difference.

### Arterial blood gas analysis

At day 10, PaO_2_ and PaCO_2_ were significantly improved in the Xuan Bai Cheng Qi group compared with PaO_2_ and PaCO_2_ in the control group (PaO_2_, FAS: mean difference 4.75, 95% CI 1.21 to 8.28, *P* = .009, PPS: mean difference 4.08, 95% IC 0.04 to 7.76, *P* = .030; PaCO_2_, FAS: mean difference -2.48, 95% CI -4.53 to -0.44, *P* = .018, PPS: mean difference -2.69, 95% CI -4.56 to -0.81, *P* = .005). There was no significant difference between the Xuan Bai Cheng Qi and control groups in PH (FAS: mean difference -0.00, 95% CI -0.01 to 0.01, *P* = .894, PPS: mean difference -0.00, 95% CI -0.01 to 0.01, *P* = .794) (Table 6).

**Table 6:** Arterial blood gas analysis in Xuan Bai Cheng Qi and control groups before and after 10-day treatment

**Variables**^**a**^	**XBCQ**^**b**^			**Control**^**b**^			**Treatment effect**^**d **^**(95% CI; *P-*** **value)**
	**Day 1 (Baseline)**	**Day 10 (End)**	**Mean D**	**Day 1 (Baseline)**	**Day 10 (End)**	**Mean D**	
PH							
FAS (122,122)	7.41 (0.05)	7.41 (0.04)	0.01 (0.05)	7.42 (0.05)	7.42 (0.05)	-0.00 (0.05)	-0.00 (-0.01, 0.01; .894)
PPS (111,118)	7.41 (0.05)	7.41 (0.04)	0.00 (0.05)	7.42 (0.05)	7.42 (0.05)	-0.00 (0.05)	-0.00 (-0.01, 0.01; .794)
PaO_2_							
FAS (122,122)	75.56 (19.38)	85.69 (16.42)	13.13 (18.12)^c^	71.47 (18.90)	80.49 (15.91)	9.01 (17.49)^c^	4.75 (1.21, 8.28; .009)
PPS (111,118)	71.91 (19.28)	85.11 (16.59)	13.20 (18.50)^c^	71.27 (19.08)	80.76 (15.96)	9.49 (17.31)^c^	4.08 (0.04, 7.76; .030)
PaCO_2_							
FAS (122,122)	47.41 (10.04)	39.52 (11.55)	-7.89 (12.27)^c^	39.52 (11.55)	41.45 (8.33)	-4.76 (8.97)^c^	-2.48 (-4.53, -0.44; .018)
PPS (111,118)	46.46 (13.32)	38.93 (9.47)	-7.53 (11.49)^c^	45.90 (11.82)	41.38 (8.41)	-4.52 (8.83)^c^	-2.69 (-4.56, -0.81; .005)

*Abbreviations*: *CI* confidence interval, *Mean D* mean difference, *PaO*_2_ arterial partial pressure of oxygen, *PaCO*_2_ arterial partial carbon dioxide pressure, *XBCQ* Xuan Bai Cheng Qi.

^a^FAS population was 122 in the XBCQ group and 122 in the control group; PPS population was 111 in the XBCQ group and 118 in the control group.

^b^Data presented as mean (SD).

^c^*P* < .01: Change between pre - treatment and post - treatment (within group difference by paired t - test).

^d^Covariance analysis estimate with 95% confidence interval for difference.

### Pro-inflammatory biomarkers (cytokines)

Only 22 participants in the Xuan Bai Cheng Qi group and 16 participants in the control group permitted their blood to be collected at the end of the trial on day 10. Therefore, comparison of biomarker levels between the two groups was based on these numbers of participants. At day 10, TNF-α, IL-4, IL-8, IL-1β, IL-6, and IL-2 levels were significantly decreased in the Xuan Bai Cheng Qi group compared with levels in the control group (Table 7).

**Table 7:** Pro-inflammatory biomarker changes in Xuan Bai Cheng Qi and control groups before and after 10 - day treatment

**Biomarkers**	**XBCQ**^**a**^**(n = 22)**			**Control**^**a**^**(n = 16)**			**Treatment effect**^**d **^**(95% CI; *P-*** **value)**
	**Day 1 (Baseline)**	**Day 10 (End)**	**Mean D**^**c**^	**Day 1 (Baseline)**	**Day 10 (End)**	**Mean D**	
TNF - α (pg/mL)	20.34 (4.90)	4.92 (1.90)	-15.42 (4.88)	18.90 (3.61)	6.47 (1.56)	-12.43 (3.28)^c^	-1.70 (-2.86, -0.54; .005)
IL - 2 (pg/mL)	129.72 (18.27)	53.50 (18.21)	-76.23 (25.21)	129.54 (28.70)	84.10 (11.01)	-45.43 (32.88)^c^	-30.60 (-40.94, -20.25; < .001)
IL - 4 (pg/mL)	5.98 (1.49)	2.36 (0.56)	-3.61 (1.78)	6.15 (1.36)	3.38 (1.25)	-2.77 (1.70)^c^	-1.02 (-1.65, -0.39; .002)
IL - 6 (pg/mL)	3.95 (0.80)	2.06 (0.53)	-1.89 (0.95)	3.81 (0.94)	2.76 (0.67)	-1.06 (0.48)^c^	-0.74 (-1.20, -0.39; < .001)
IL - 8 (pg/mL)	18.33 (3.93)	9.09 (3.54)	-9.24 (6.11)	18.01 (4.17)	14.92 (3.86)	-3.08 (5.36)^b^	-5.80 (-8.26, -3.34; < .001)
IL - 1β (pg/mL)	13.42 (2.12)	8.36 (1.02)	-7.89 (12.27)	12.86 (1.98)	9.23 (1.54)	-4.76 (8.97)^c^	-0.94 (-1.78, -0.10; .029)

*Abbreviations*: *CI* confidence interval, *IL* *- 1β* interleukin - 1 beta, *IL - 2* interleukin - 2, *IL - 4* interleukin - 4, *IL - 6* interleukin - 6, *IL - 8* interleukin - 8, *Mean D* mean difference, *TNF - α* tumor necrosis factor alpha, *XBCQ* Xuan Bai Cheng Qi.

^a^Data presented as mean (SD).

^b^*P* < .05.

^c^*P* < .01: change between pre - treatment and post - treatment (within group difference by paired t - test).

^d^Covariance analysis estimate with 95% confidence interval for difference.

### Oxidant/antioxidant balance

Based on serum collected from the 20 participants in the Xuan Bai Cheng Qi group and 20 participants in the control group, MDA level was significantly decreased in the Xuan Bai Cheng Qi group compared with that in the control group (mean difference -1.07, 95% CI -1.55 to -0.59, *P* < .001), while SOD level in the Xuan Bai Cheng Qi group was significantly higher than that in the control group (mean difference 6.09, 95% CI 2.94 to 9.23, *P* < .001) (Table 8).

**Table 8:** Serum SOD and MDA in Xuan Bai Cheng Qi and control groups before and and after treatment

**Variables**	**XBCQ**^**a**^**(n = 20)**			**Control**^**a**^**(n = 20)**			**Treatment effect**^**c **^**(95% CI; *P *** **value)**
	**Day 1 (Baseline)**	**Day 10 (End)**	**Mean D**^**b**^	**Day 1 (Baseline)**	**Day 10 (End)**	**Mean D**	
SOD (U/mL)	20.56 (4.95)	28.54 (4.66)	7.98 (5.97)	21.90 (5.32)	23.24 (6.76)	1.33 (4.21)	6.09 (2.94, 9.23; < .001)
MDA (nmol/mL)	4.43 (1.52)	1.89 (0.52)	-2.54 (1.65)	4.52 (1.44)	2.98 (1.45)	-1.54 (0.94)^b^	-1.07 (-1.55, -0.59; < .001)

*Abbreviations*: *CI* confidence interval, *MDA* malondialdehyde, *Mean D* mean difference, *SOD* superoxide dismutase, *XBCQ* Xuan Bai Cheng Qi.

^a^Data presented as mean (SD).

^b^*P* < .01: Change between pre-treatment and post-treatment (within group difference by paired t-test).

^c^Covariance analysis estimate with 95% confidence interval for difference.

### Safety variables

Adverse events were recorded during the study period. Mild diarrhea occurred in six participants (4.92%) in the Xuan Bai Cheng Qi group and in two participants (1.64%) in the control group, but these incidents were not statistically significant (*P* = .281). There were no significant differences in blood, urine, and liver and kidney function tests, or in electrocardiography results before and after the study period between the Xuan Bai Cheng Qi and control groups (Table 9).

**Table 9:** Safety profile

**Variables**^**a**^	**XBCQ**^**b**^**(n = 122)**		**Control**^**b**^**(n = 122)**		***P-*** **value**^**a**^
	**Day 1 (Baseline)**	**Day 10 (End)**	**Day 1 (Baseline)**	**Day 10 (End)**	
WBC (10^9^/L)	8.33 (3.49)	7.51 (2.81)	9.38 (7.95)	7.53 (3.11)	.843
HB (g/L)	138.2 (24.0)	138.6 (31.0)	136.3 (28.1)	134.3 (28.1)	.310
RBC (10^12^/L)	4.39 (0.65)	4.38 (0.64)	4.33 (0.61)	4.27 (0.57)	.263
PLT (10^9^/L)	198.4 (62.4)	210.4 (78.7)	209.4 (76.9)	207.5 (68.4)	.178
NEUT (%)	69.6 (14.4)	64.7 (12.9)	68.9 (14.9)	62.1 (13.4)	.111
ALT (U/L)	21.0 (11.1)	22.2 (12.1)	21.9 (16.3)	22.5 (14.1)	.920
BUN (mmol/L)	5.6 (1.6)	5.7 (2.4)	6.1 (2.8)	5.8 (2.8)	.511
CR ( μmmol/L)	75.4 (20.4)	74.0 (21.5)	85.2 (46.5)	80.3 (35.9)	.863
BUN (n)^c^	27 (122)	32 (122)	15 (122)	7 (122)	.116
ECG (n)^c^	56 (122)	45 (122)	57 (122)	47 (122)	.895

*Abbreviations*: *ALT* alanine aminotransferase, *BUN* blood urea nitrogen, *Cr* creatinine, *ECG* electrocardiogram, *HB* hemoglobin, *NEUT* neutrophilic granulocyte, *PLT* platelets, *RBC* red blood cell, *WBC* white blood cell.

^a^Covariance analysis for between group after treatment. ^b^Data presented as mean (SD).

^c^Chi-square test for between group after treatment.

### Adherence

Adherence based on empty granule package count was high and similar in both the Xuan Bai Cheng Qi and control groups.

## Discussion

This multicenter, randomized, double-blind, placebo-controlled study showed that as an adjuvant treatment Xuan Bai Cheng Qi formula produced a significant improvement in symptoms associated with AECOPD of the TCM syndrome type, phlegm-heat obstructing the lungs. Total symptom scores from baseline showed that Xuan Bai Cheng Qi ameliorated cough, phlegm, wheezing, and chest congestion. Lung function and blood gas levels were also significantly improved in the intervention group compared with the control group.

AECOPD appears to be triggered by increased airway inflammation. A number of pro-inflammatory cytokines, such as TNF-α, IL-4, IL-8, IL-1β, IL-6, IL-2, have been detected during COPD exacerbation and have been reported to be increased in both sputum and serum during COPD exacerbations [21-25]. In our study, levels of pro-inflammatory mediators in the Xuan Bai Cheng Qi group were lower than those in the control group. We propose that as an adjuvant treatment, Xuan Bai Cheng Qi contributed to regulating systemic inflammatory response. Previous studies have shown that the active ingredients in the herbs that comprise Xuan Bai Cheng Qi formula, such as emodin in *Rheum officinale* Baill [26], calcium sulfate in Gypsum fibrosum [27], triterpenoid saponins in *Trichosanthes kirilowii* Maxim [28]*,* and amygdalin in Semen *Armeniacae amarum*[29] possess anti-inflammatory effects.

Oxidative stress is another component in the pathogenesis of AECOPD [30-32]. Oxidative stress can induce extensive tissue damage and activate the nuclear factor kappa-light-chain-enhancer of activated B cells (NF-κB) pathway, which provokes the release of pro-inflammatory molecules, such as TNF-α, IL-8. Our results indicate that compared with the control group, SOD activity was higher and MDA content was lower in the Xuan Bai Cheng Qi group. Thus Xuan Bai Cheng Qi may contribute to restoring oxidant/antioxidant balance.

Mention needs to be made that of the 122 participants each in the Xuan Bai Chang Qi and control groups, only 20 and 16 participants, respectively, allowed their serum to be collected at the end of the trial for evaluation of biomarker changes. A similar situation occurred during assessment of levels of SOD and MDA, with only 20 participants in each group permitting serum collection. For ethical considerations, we respected participants’ inclination. Nevertheless, based on the results we did obtain, there were significant differences between the two groups as described above.

Xuan Bai Cheng Qi formula appears to be safe. Serious untoward effects were not reported during the study. Six participants in the Xuan Bai Cheng Qi group experienced mild diarrhea. Toxicological studies on the active ingredient in individual medicinals in Xuan Bai Cheng Qi formula have shown that long-term use of *Rheum officinale* Baill may induce liver degeneration [33]; toxicity of Semen *Armeniacae amarum* is mainly from amygdaloside, which can produce the toxic hydrocyanic acid by enzymatic hydrolysis [34]; *Trichosanthes kirilowii* Maxim is not known to be toxic [35]; and Gypsum fibrosum is mildly toxic [36]. Typically, during processing of raw medicinals and decoction of the combined medicinals, it is likely that the individual toxic effects are reduced or even neutralized [37].

The main limitation of this study is its short duration of 10 days. Changes in frequency of acute exacerbations of COPD were not observable. In addition, the comparison between biomarkers and levels of SOD and MDA are of an observational nature, which may therefore be biased. Further studies should be performed to evaluate the long-term efficacy of Xuan Bai Cheng Qi formula as an adjuvant treatment for AECOPD.

## Conclusions

Traditional Chinese medicinals are commonly used to treat lung conditions, including acute exacerbation of chronic obstructive pulmonary disease (AECOPD). However, there is a lack of modern research to validate such empirical remedies. To the best of our knowledge, this study is the first large-scale, multicenter randomized control trial evaluating the efficacy and safety in humans of the traditional formula Xuan Bai Cheng Qi as an adjuvant treatment for AECOPD of the TCM syndrome type, phlegm-heat blocking the lungs. Our results found that compared with placebo, Xuan Bai Cheng Qi appears to be safe and effective for AECOPD with improvement in symptoms as well as biomarker parameters.

## Abbreviations

COPD: Chronic obstructive pulmonary disease; TCM: Traditional Chinese medicine; AECOPD: Acute exacerbation of chronic obstructive pulmonary disease; FVC: Forced vital capacity; FEV_1_: Forced expiratory volume in one second; FEV_1_%pred: FEV_1_ percentage of predicted value; SOD: Superoxide dismutase; MDA: Malondialdehyde.

## Competing interests

The authors declare that they have no competing interests.

## Authors’ contributions

YL and XZ conceived and designed the study. RW, YS, and JZ input the data, and ML and FZ analysed the data and drafted the manuscript. All authors read and approved the final manuscript.

## Pre-publication history

The pre-publication history for this paper can be accessed here:

http://www.biomedcentral.com/1472-6882/14/239/prepub
